# Nonlinear Association of Length of Stay with In-Hospital Mortality in Alzheimer’s Disease Hospitalizations: Admission-Only vs. Inpatient-Course Prediction Using Explainable Machine Learning

**DOI:** 10.3390/geriatrics11050136

**Published:** 2026-09-17

**Authors:** Tursun Alkam, Ebrahim Tarshizi, Andrew H. Van Benschoten

**Affiliations:** Program of Applied Artificial Intelligence, University of San Diego, San Diego, CA 92110, USA

**Keywords:** Alzheimer’s disease, length of stay, in-hospital mortality, hospitalization, machine learning, explainable artificial intelligence, SHAP

## Abstract

**Background:** Hospitalizations among patients with Alzheimer’s disease (AD) carry substantial mortality risk, but length of stay (LOS) is time-dependent and may reflect heterogeneous inpatient trajectories. We examined unadjusted and adjusted LOS–mortality patterns and compared admission-only versus inpatient-course prediction using explainable machine learning. **Methods:** Using the full 2017 Nationwide Readmissions Database (NRD), we identified hospitalizations among adults aged ≥60 years with an ICD-10-CM G30.x AD code in any diagnosis position. Records with missing in-hospital mortality status were excluded. LOS was summarized in clinically interpretable bins and modeled using restricted cubic splines. Model A excluded explicit inpatient-course measures, whereas Model B added LOS, procedure count, and total charges. Performance was evaluated using patient-grouped 5-fold out-of-fold validation and summarized by AUROC and AUPRC; SHAP was used for interpretation. **Results:** Among 249,507 AD hospitalizations, 12,666 in-hospital deaths occurred (5.08%; weighted mortality 4.97%). Unadjusted mortality was highest at LOS 0–1 day (13.00%), lowest at 4–6 days (3.47%), and increased to 7.77% at ≥22 days. After multivariable adjustment, LOS remained strongly nonlinear, but adjusted predicted mortality declined across the modeled LOS range. Model A achieved AUROC/AUPRC of 0.780/0.180, whereas Model B improved to 0.828/0.329. Sepsis, diagnostic burden, acute kidney injury, age, stroke, and pneumonia were stable predictors; LOS and procedure burden added prognostic information in Model B. **Conclusions:** The crude LOS–mortality pattern was U-shaped, whereas the adjusted pattern suggests that the late-stay increase in unadjusted mortality is partly explained by patient complexity and evolving inpatient-course factors. Admission-only prediction provides meaningful early risk stratification, while inpatient-course information improves prognostic assessment as hospitalization evolves.

## 1. Introduction

In the inpatient setting, AD commonly coexists with frailty, polypharmacy, functional dependence, and reduced physiologic reserve, which can amplify the effects of infection, hemodynamic instability, acute kidney injury, respiratory failure, and cerebrovascular events [1,2,3,4]. Older adults with dementia are also particularly susceptible to delirium during acute illness and hospitalization, and delirium superimposed on chronic cognitive impairment can complicate assessment, treatment, and recovery [5,6]. Consequently, hospitalizations involving AD are often clinically complex and carry meaningful mortality and care-coordination burdens [4,7,8,9].

Length of stay (LOS) is widely used as a pragmatic marker of hospital resource use and complexity, and systematic review evidence indicates that patients with dementia often experience longer hospitalizations than patients without dementia [7]. LOS, however, is fundamentally time-dependent and may reflect competing trajectories rather than a simple monotonic measure of severity. Very early death can truncate LOS, whereas prolonged hospitalization may reflect organ dysfunction, complications, delayed recovery, or discharge barriers [10,11,12,13]. Studies in other hospitalized populations have also shown that both unusually short and prolonged stays may be associated with adverse outcomes [14]. These considerations make flexible modeling essential when LOS is examined in relation to mortality.

In parallel, predictive modeling of inpatient outcomes has advanced through methods that can capture nonlinearities and interactions among clinical factors [12,15,16]. In dementia research, much of the literature has focused on hospitalization risk, long-term prognosis, or single-system cohorts [17,18,19]. A further challenge is temporal interpretability: models may combine information plausibly available early in hospitalization with variables that accumulate during the inpatient course, such as LOS, procedures, and hospital charges. Prediction-reporting frameworks emphasize the importance of defining the intended prediction time and avoiding leakage from downstream information [20,21,22].

Using the 2017 NRD, we examined adults aged ≥60 years with an AD diagnosis in any diagnosis field. We first characterized the unadjusted LOS–mortality relationship using clinically interpretable categories and then modeled LOS flexibly with restricted cubic splines. We next contrasted Model A, which excluded explicit inpatient-course measures, with Model B, which incorporated LOS, procedure count, and total charges. This framework was designed to distinguish risk information available without explicit course proxies from additional prognostic information that emerges as hospitalization progresses.

By pairing a trajectory-informed LOS analysis with patient-grouped machine learning evaluation and fold-level SHAP stability assessment, the study aims to provide a transparent view of mortality risk in AD hospitalizations. The analysis also examines diagnosis family patterns at LOS extremes to place the statistical findings in clinical context and to identify complications that may characterize prolonged hospital courses.

## 2. Methods

### 2.1. Data Source and Study Design

We conducted this study using the 2017 Healthcare Cost and Utilization Project (HCUP) Nationwide Readmissions Database (NRD), sponsored by the Agency for Healthcare Research and Quality [23]. The NRD is an all-payer, calendar year inpatient discharge database constructed from State Inpatient Databases with verified patient linkage identifiers. The 2017 NRD was assembled from 28 participating states and community hospitals, excluding rehabilitation and long-term acute-care hospitals. It contains approximately 18 million unweighted discharges and represents approximately 35.8 million U.S. discharges after weighting. Patient linkage identifiers permit hospitalizations for the same individual to be grouped across hospitals within a participating state during the calendar year. Discharge-level analyses incorporated the NRD discharge weight (DISCWT) to generate national estimates where appropriate. The 2017 database was selected as a complete pre-pandemic national baseline with full-year ICD-10-CM/PCS coding and patient linkage suitable for leakage-resistant, grouped validation.

### 2.2. Study Population and Alzheimer’s Disease Cohort Definition

We restricted the analysis to hospitalizations among adults aged ≥60 years and required a documented AD diagnosis in any available diagnosis position. Qualifying ICD-10-CM codes were G30.0 (AD with early onset), G30.1 (AD with late onset), G30.8 (other AD), and G30.9 (AD, unspecified). A qualifying G30.x code could occur in the principal or any secondary diagnosis field. Broader dementia or neurocognitive disorder codes, including F01, F02, F03, or mild cognitive impairment/neurocognitive disorder codes, were not used as substitutes for a G30.x diagnosis. Thus, the cohort was intentionally AD-specific rather than an all-cause dementia cohort. Hospitalizations could still contain additional dementia-related codes if G30.x was also documented. Records with missing in-hospital mortality status were excluded from the primary analytic cohort, and records with missing LOS were excluded only from LOS-dependent analyses.

### 2.3. Outcome

The primary outcome was in-hospital mortality, defined using the NRD discharge indicator for death during hospitalization (DIED; binary). Missing DIED values were treated as having unknown outcome status and were excluded rather than recoded as survival.

### 2.4. Predictors

Covariates were selected a priori based on clinical relevance and availability in the NRD. Demographic and admission context variables included age, sex, primary payer (PAY1), ZIP Code income quartile (ZIPINC_QRTL), metropolitan/non-metropolitan classification (PL_NCHS), weekend admission (AWEEKEND), elective admission status (ELECTIVE), and emergency department (ED) involvement. HCUP_ED was collapsed to a binary indicator defined as any evidence of ED services (HCUP_ED values 1–4) versus no evidence of ED services (HCUP_ED = 0) to avoid sparse administrative subcategories. Clinical severity and comorbidity markers included diagnostic burden (I10_NDX) and indicators for sepsis, acute kidney injury, stroke, congestive heart failure, chronic kidney disease, urinary tract infection, pneumonia, delirium, and chronic obstructive pulmonary disease, identified from ICD-10-CM diagnosis families. Inpatient-course measures were LOS, procedure burden (I10_NPR), and total hospital charges (TOTCHG). Total charges were scaled per $10,000 for regression analyses.

### 2.5. Analytic Strategy and Prespecified Two-Model Design

To separate explicit hospitalization-course measures from the remaining feature set, we prespecified two complementary models. Model A (“admission-only”) included demographics, admission context, diagnostic burden, and clinical condition indicators but excluded LOS, procedure count, and total charges. Model B (“full inpatient course”) added these three course variables. The same feature definitions were used across regression, machine learning, and explainability analyses. Because diagnosis indicators in the NRD are discharge-level codes without reliable timestamping for every condition, the term “admission-only” refers specifically to exclusion of explicit inpatient-course utilization measures and should not be interpreted as proof that every coded diagnosis was known at the moment of admission.

### 2.6. Statistical Analyses

Patient and hospitalization characteristics were summarized overall and by in-hospital death status. Weighted means, weighted counts, and weighted percentages were used for national descriptive estimates; *p*-values comparing survivors and decedents were obtained using unweighted two-sample tests, with a two-sided Wilcoxon rank-sum test for LOS because of its skewed distribution. LOS was categorized as 0–1, 2–3, 4–6, 7–9, 10–14, 15–21, and ≥22 days, and unadjusted mortality with 95% binomial confidence intervals was calculated within each category (Supplementary Table S1). Multivariable associations with mortality were estimated using weighted logistic regression corresponding to Models A and B. In Model B, LOS was modeled using restricted cubic splines with 5 degrees of freedom. The spline terms were assessed jointly with a Wald test, and nonlinearity was additionally evaluated against a linear LOS specification by likelihood-ratio testing. Sensitivity analyses varying spline degrees of freedom from 4 to 6 are reported in Supplementary Table S2. Adjusted odds ratios (ORs) with 95% confidence intervals (CIs) were reported. Robust variance specifications, including hospital-clustered alternatives, were examined as sensitivity analyses.

### 2.7. Machine Learning, Model Evaluation, and Explainability

Gradient-boosted decision trees (XGBoost) were trained under the same Model A and Model B feature sets. Continuous variables were median-imputed, categorical variables were most-frequent imputed and one-hot encoded, and the primary evaluation used patient-grouped 5-fold cross-validation based on NRD_VisitLink. Each hospitalization received an out-of-fold prediction from a model that had not been trained on any hospitalization from the same linked patient. Primary discrimination was calculated from pooled out-of-fold predictions using AUROC and AUPRC; fold-level values were retained to assess stability. Threshold-dependent precision, recall, and F1 metrics are provided in Supplementary Table S3. A regularized logistic regression comparator was evaluated with the same grouped folds. SHapley Additive exPlanations (SHAP) were calculated on held-out fold data, and feature stability was summarized by frequency and average rank among the top features across folds (Supplementary Table S4). To facilitate comparison, predictors shared by Models A and B were displayed in a common order across panels.

### 2.8. Sensitivity Analyses

To determine whether the LOS–mortality association was driven predominantly by early-death or rapid-discharge hospitalizations, adjusted spline analyses were repeated after excluding LOS ≤ 1 day and, separately, LOS ≤ 2 days.

### 2.9. Decision Curve Analysis

Clinical utility was evaluated using decision curve analysis based on the same patient-grouped, out-of-fold probabilities. Net benefit for Models A and B was compared with treat-all and treat-none strategies across threshold probabilities from 0.01 to 0.10 (Supplementary Figure S1; Supplementary Table S5).

### 2.10. Diagnosis Family Analysis at LOS Extremes

To contextualize LOS extremes, we compared three-character ICD-10-CM diagnosis family prevalence between short LOS (≤5 days) and prolonged LOS (≥25 days) hospitalizations. Each diagnosis family was counted at most once per hospitalization. The cohort-defining AD family (G30), dementia families F01-F03, Z-code families, and external-cause families beginning with V, W, X, or Y were excluded so that the tables emphasized clinical diagnoses rather than cohort-defining, administrative, or external-cause codes. Supplementary Table S6A,B shows the leading diagnosis families in each LOS group, and Supplementary Table S6C ranks families by absolute prevalence enrichment in prolonged versus short stays.

### 2.11. Data Availability

The 2017 NRD is a restricted HCUP dataset. Discharge-level data cannot be publicly shared under the HCUP Data Use Agreement. Researchers may obtain the database directly from HCUP after completing the required training and executing the applicable Data Use Agreement. All analyses use de-identified administrative discharge data.

### 2.12. Software and Code Availability

Analyses were conducted in Python 3.12.13 using statsmodels 0.14.6, XGBoost 3.3.0, SHAP 0.52.0, scikit-learn 1.6.1, pandas 2.2.2, NumPy 2.0.2, SciPy 1.16.3, and patsy 1.0.2., and related scientific-computing libraries. The revised analysis code used to define the G30.x cohort, generate tables and figures, fit regression and machine learning models, perform grouped validation, and calculate explainability outputs is available at https://github.com/TAlkam/NRD_2017 (accessed on 14 September 2026). 

## 3. Results

### 3.1. Study Cohort and In-Hospital Mortality

The full G30.x, age ≥ 60 cohort contained 249,633 hospitalizations. After exclusion of 126 hospitalizations with missing DIED status, the final analytic cohort included 249,507 hospitalizations, with 12,666 in-hospital deaths (unweighted mortality 5.08%; Table 1). After applying NRD discharge weights, the cohort represented an estimated 455,256 U.S. hospitalizations and 22,612 deaths (weighted mortality 4.97%). Sixty-nine hospitalizations had missing LOS and were omitted only from LOS-dependent analyses, leaving 249,438 hospitalizations for LOS analyses. Compared with survivors, decedents were older (weighted mean 83.95 vs. 82.39 years, *p* < 0.001), had greater diagnostic burden (20.90 vs. 17.06 diagnoses, *p* < 0.001), and underwent more procedures (1.83 vs. 0.86, *p* < 0.001). Weighted median LOS was 4 days in both groups, but the distributions differed (survivors 4 [IQR 3–7] vs. decedents 4 [IQR 2–9], *p* < 0.001).

### 3.2. Unadjusted Relationship Between Length of Stay and Mortality

**Figure 1:**
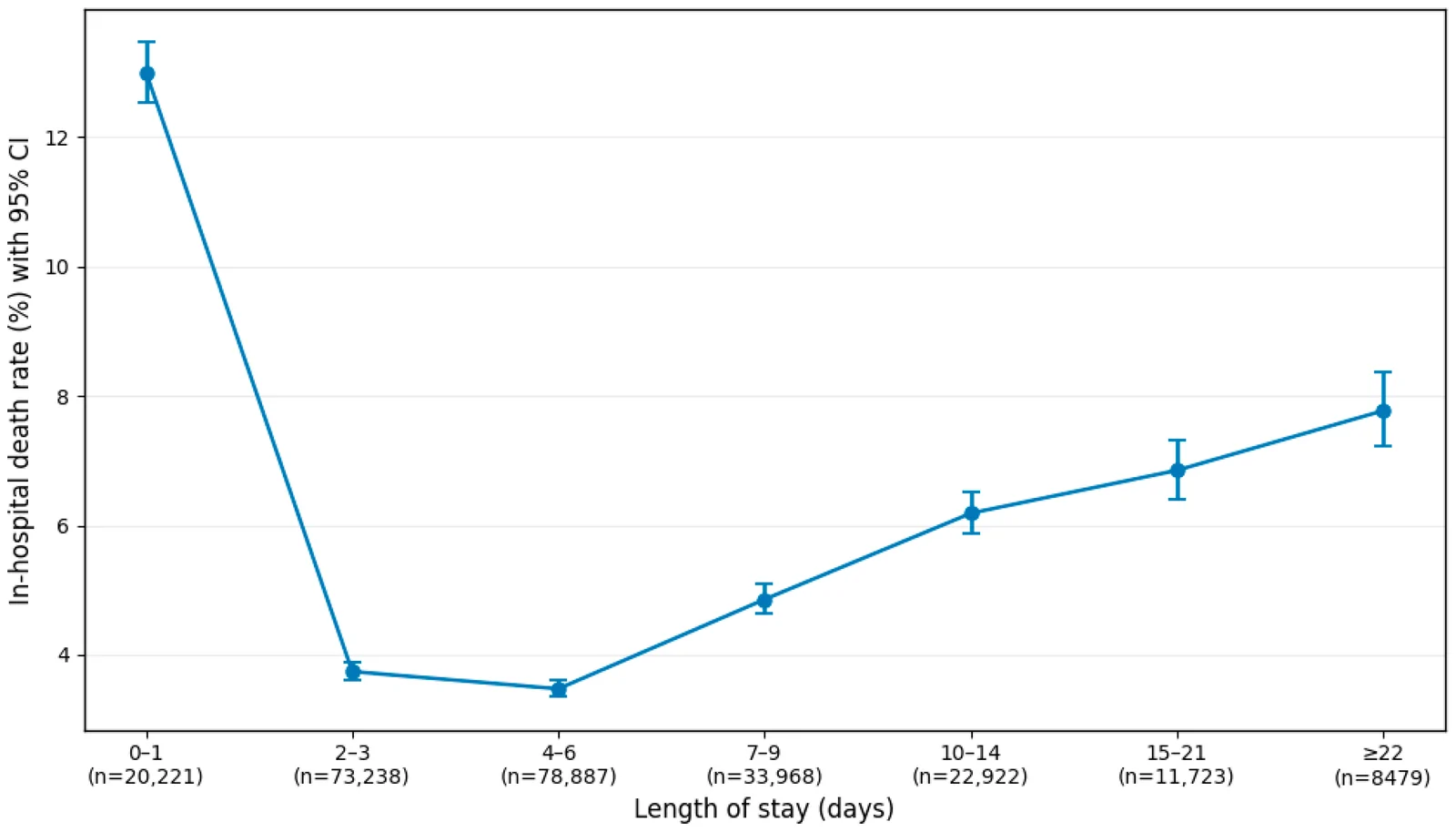
Unadjusted association between length of stay and in-hospital mortality in Alzheimer’s disease hospitalizations. Observed in-hospital mortality rates are shown across clinically defined length-of-stay (LOS) categories among adults aged ≥60 years hospitalized with documented Alzheimer’s disease in the 2017 Nationwide Readmissions Database. Points represent the percentage of hospitalizations ending in in-hospital death, and error bars indicate 95% confidence intervals. The number of hospitalizations in each LOS category is shown below the x-axis. Mortality was highest for LOS 0–1 days, declined to its lowest level at 4–6 days, and increased again with prolonged hospitalization, demonstrating an unadjusted U-shaped LOS–mortality pattern. Hospitalizations with missing LOS were excluded from this analysis.

The unadjusted LOS–mortality relationship was U-shaped across clinically interpretable LOS categories (Figure 1; Supplementary Table S1). Mortality was highest for LOS 0–1 day (13.00%, 95% CI 12.54–13.47), declined to its lowest level at 4–6 days (3.47%, 95% CI 3.35–3.60), and then increased with longer hospitalization, reaching 6.85% at 15–21 days and 7.77% (95% CI 7.22–8.36) at ≥22 days. Hospitalizations lasting 0–1 day represented 8.11% of LOS-complete admissions but accounted for 20.76% of all deaths in the LOS-complete cohort. 

### 3.3. Multivariable Regression Analyses for In-Hospital Death

In weighted Model A, the strongest clinical associations with in-hospital mortality included sepsis (OR 3.61, 95% CI 3.50–3.73), stroke (OR 2.75, 95% CI 2.61–2.90), acute kidney injury (OR 1.90, 95% CI 1.84–1.96), pneumonia (OR 1.55, 95% CI 1.49–1.60), and congestive heart failure (OR 1.33, 95% CI 1.28–1.37), all *p* < 0.001 (Table 2). Older age (OR 1.04 per year) and greater diagnostic burden (OR 1.05 per additional diagnosis) were also independently associated with mortality. Urinary tract infections and chronic kidney disease showed lower adjusted odds within the full covariate context.

In Model B, the core clinical associations remained similar: sepsis OR 3.59, stroke OR 2.84, acute kidney injury OR 1.90, and pneumonia OR 1.60 (all *p* < 0.001). Procedure burden was independently associated with mortality (OR 1.18 per additional procedure, 95% CI 1.17–1.19; *p* < 0.001), whereas total charges were not independently significant after adjustment (*p* = 0.459). LOS spline terms were strongly associated with mortality overall (Wald *p* = 3.90 × 10^−187^), and the restricted cubic spline specification fit significantly better than a linear LOS term (likelihood-ratio *p* = 2.43 × 10^−256^), confirming substantial nonlinearity.

### 3.4. Explainable Machine Learning Performance and Feature Interpretation

**Figure 2:**
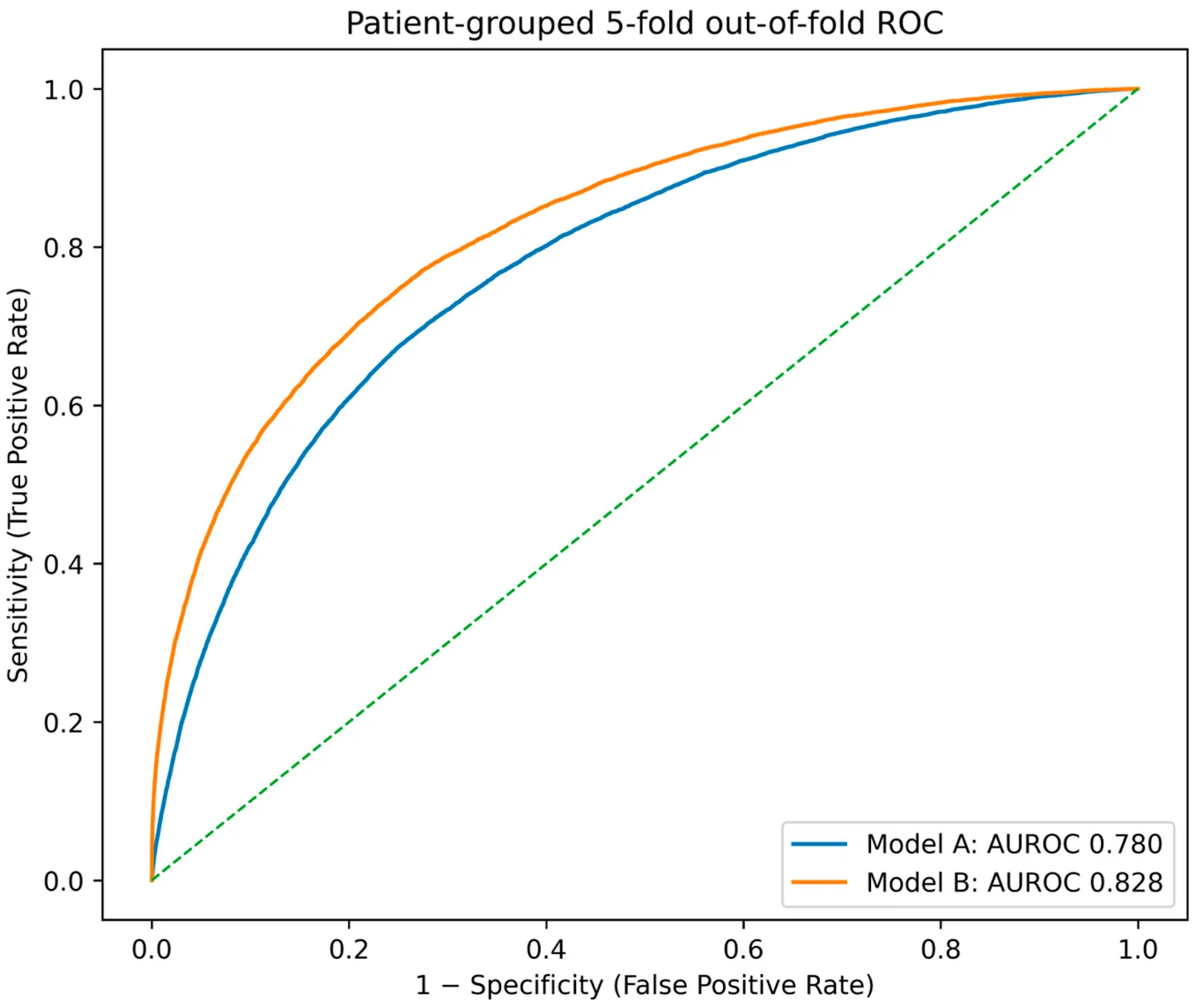
Patient-grouped 5-fold out-of-fold receiver operating characteristic curves for prediction of in-hospital mortality. Receiver operating characteristic (ROC) curves compare the admission-oriented Model A with the full inpatient-course Model B. Five-fold cross-validation was performed with patient-level grouping by NRD_VisitLink so that hospitalizations from the same patient could not appear in both training and validation sets. In each fold, approximately 80% of unique patient groups were used for model development and 20% for validation, and performance was evaluated using pooled out-of-fold predictions. Model A achieved an area under the ROC curve (AUROC) of 0.780, whereas Model B achieved an AUROC of 0.828. The diagonal dashed line represents discrimination expected by chance. Model A excludes explicit inpatient-course measures, whereas Model B additionally includes length of stay, procedure count, and total hospital charges. Five-fold cross-validation was selected to provide a practical balance among training-set size, reliable out-of-fold performance estimation, and computational efficiency in this large national dataset.

Using pooled patient-grouped out-of-fold predictions, Model A achieved AUROC 0.780 and AUPRC 0.180, whereas Model B achieved AUROC 0.828 and AUPRC 0.329 (Figure 2; Supplementary Table S3). Fold-level AUROC variability was low (SD 0.001 for Model A and 0.004 for Model B), supporting stability across patient-grouped splits. Under the same evaluation, the regularized logistic comparator achieved AUROC/AUPRC of 0.776/0.175 for Model A and 0.784/0.193 for Model B (Supplementary Table S7).

**Figure 3:**
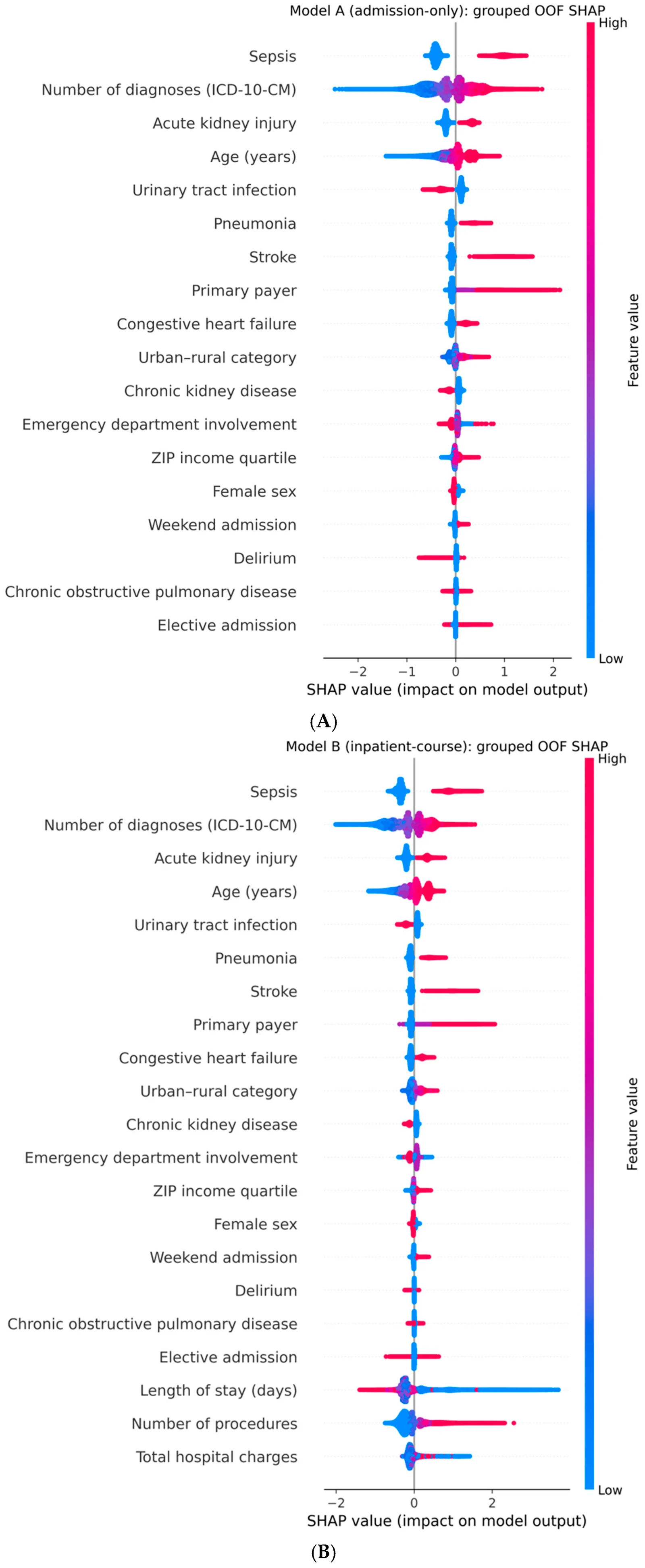
SHAP-based interpretation of the admission-oriented and inpatient-course machine learning models for in-hospital mortality. (**A**) SHapley Additive exPlanations (SHAP) summary plot for Model A, which excludes explicit inpatient-course variables. (**B**) SHAP summary plot for Model B, which additionally includes length of stay, procedure count, and total hospital charges. Each point represents a hospitalization, and the horizontal position indicates the SHAP value, representing the contribution of that feature to the model output for that hospitalization. Positive SHAP values indicate contributions toward higher predicted mortality risk, whereas negative values indicate contributions toward lower predicted risk. Color represents the feature value, with red indicating higher values and blue indicating lower values; for binary features, this corresponds to presence versus absence. Shared predictors are displayed in the same vertical order across panels to facilitate direct comparison. SHAP values represent model attribution and should not be interpreted as causal effects.

SHAP analysis showed highly stable feature rankings across folds (Figure 3A,B; Supplementary Table S4). In Model A, sepsis, diagnostic burden, acute kidney injury, age, urinary tract infection, pneumonia, and stroke were recurrent high-impact features. In Model B, sepsis and diagnostic burden remained dominant, while LOS and procedure count became prominent course-related contributors. Total charges did not appear among the most stable top-ranked Model B features. Shared predictors are presented in a common vertical order across Figure 3 panels to facilitate direct comparisons.

### 3.5. Robustness and Sensitivity Analyses

**Figure 4:**
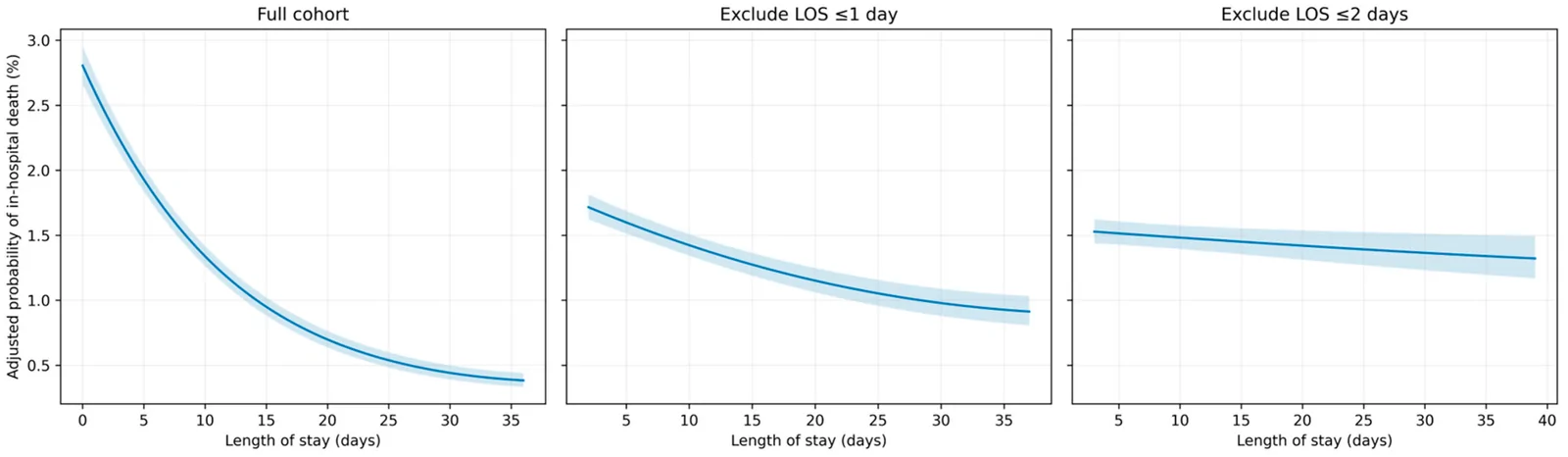
Adjusted nonlinear association between length of stay and in-hospital mortality with sensitivity analyses excluding very short stays. Adjusted predicted probabilities of in-hospital mortality across length of stay (LOS) were estimated using multivariable logistic regression with LOS modeled by restricted cubic splines. The (**left**) panel shows the full LOS-complete cohort, the (**middle**) panel excludes hospitalizations with LOS ≤ 1 day, and the (**right**) panel excludes hospitalizations with LOS ≤ 2 days. Solid lines represent adjusted predicted mortality probabilities and shaded bands represent 95% confidence intervals. LOS remained significantly nonlinear after multivariable adjustment, but adjusted predicted mortality declined across the modeled LOS range rather than reproducing the U-shaped pattern observed in the unadjusted analysis. These sensitivity analyses demonstrate that the adjusted LOS–mortality relationship was not driven solely by very short hospitalizations.

Among the 249,438 hospitalizations with non-missing LOS, 12,635 deaths occurred (5.07%). Mortality decreased to 4.37% after excluding LOS ≤ 1 day (10,006 deaths among 229,217 hospitalizations) and to 4.27% after excluding LOS ≤ 2 days (8472 deaths among 198,246 hospitalizations), confirming the disproportionate contribution of very short stays to mortality. In adjusted restricted cubic spline analyses, LOS remained nonlinear in the full cohort and both sensitivity samples; however, adjusted predicted mortality declined across the modeled LOS range rather than reproducing the late-stay increase seen in the unadjusted categories (Figure 4). Thus, the U-shaped pattern was descriptive at the crude level, whereas adjustment materially changed the shape of the association.

### 3.6. Clinical Diagnosis Family Patterns in Short Versus Prolonged Length of Stay

Diagnosis family profiles differed substantially between short and prolonged hospitalizations (Supplementary Table S6A–C). Among short LOS admissions (≤5 days; N = 152,991), common diagnoses included hyperlipidemia (46.61%), hypertension (44.31%), type 2 diabetes (29.43%), fluid/electrolyte disorders (29.20%), chronic ischemic heart disease (27.58%), chronic kidney disease (25.21%), atrial fibrillation/flutter (24.62%), urinary tract infection (24.56%), and acute kidney injury (22.53%).

Among prolonged LOS admissions (≥25 days; N = 6212), hypertension (48.39%) and fluid/electrolyte disorders (46.28%) remained common, while urinary tract infection (34.63%), acute kidney injury (31.13%), respiratory failure (20.81%), sepsis (19.59%), dysphagia (19.96%), and delirium (16.65%) were more prominent. The largest prevalence enrichments in prolonged versus short stays included fluid/electrolyte disorders (+17.08 percentage points), dysphagia (+11.88), delirium (+11.43), urinary tract infection (+10.07), respiratory failure (+9.80), and acute kidney injury (+8.61). Pressure ulcers were present in 13.67% of prolonged stays versus 5.66% of short stays, an 8.01-percentage-point difference (prevalence ratio 2.41).

### 3.7. Decision Curve Analysis

Across the evaluated threshold range of 0.01–0.10, both models provided greater net benefit than the treat-all and treat-none strategies, and Model B consistently provided greater net benefit than Model A (Supplementary Figure S1; Supplementary Table S5).

## 4. Discussion

This national analysis provides two complementary insights. First, the crude relationship between LOS and in-hospital mortality is distinctly U-shaped, with mortality concentrated among very short stays and rising again in prolonged stays. Second, the late-stay increase is not preserved after multivariable adjustment: adjusted mortality remains strongly nonlinear but declines across the observed LOS range. This distinction changes the interpretation of LOS from an apparent independent late-stay risk marker to a time-dependent summary of competing clinical processes. The admission-versus-course modeling framework further shows that clinically meaningful risk information is available without explicit course measures, while LOS and procedure burden add prognostic information as hospitalization evolves.

### 4.1. LOS as a Trajectory Marker: Crude and Adjusted Patterns

The unadjusted U-shaped pattern is consistent with two recognizable hospitalization trajectories. Very short stays can reflect catastrophic illness, rapid deterioration, or care transitions that truncate LOS; by contrast, prolonged stays often accumulate organ dysfunction, complications, and discharge barriers. Prior dementia literature has generally emphasized longer LOS and worse hospital outcomes rather than the shape of the LOS–mortality association [7,17,18,19]. Methodological work on LOS and mortality cautions that death competes with discharge and can make LOS appear protective or harmful depending on how time and survivorship are handled [13]. Our adjusted results reinforce that caution: once clinical and course-related factors were included, the late crude mortality rise was attenuated rather than reproduced. Accordingly, LOS should be interpreted as a trajectory marker, not a causal exposure.

Recent studies provide useful contemporary context. De Matteis et al. identified acute illness and multimorbidity as important correlates of in-hospital mortality among older patients with dementia [24]. Di Martino et al., using administrative admissions from 2018 to 2023, reported that dementia was associated with both in-hospital mortality and prolonged LOS after propensity score matching [9]. These post-2017 findings are directionally consistent with the vulnerability seen in our cohort, but direct numerical comparisons are inappropriate because populations, coding systems, healthcare settings, and the COVID-19 period differ. The present NRD analysis should therefore be viewed as a large pre-pandemic U.S. baseline rather than a direct estimate of current practice.

### 4.2. Clinical Drivers of Mortality in the Context of Prior Evidence

Sepsis, acute kidney injury, stroke, pneumonia, older age, and greater diagnostic burden were consistently prominent across regression and SHAP analyses. These signals fit the broader dementia hospitalization literature, in which frailty and limited physiologic reserve magnify the consequences of infection, organ failure, and neurologic insults [4,17,24]. The stability of these predictors across methods is more informative than the magnitude of any single coefficient because administrative diagnosis coding is influenced by documentation practices and clinical workup. The inverse adjusted association observed for urinary tract infection should therefore not be interpreted as a protective effect; it may reflect competing severity, coding intensity, or the distinction between localized infection and more severe systemic illness.

A further consideration is under-recognition of cognitive disease in hospital records. Many older adults with dementia remain undiagnosed or unaware of their diagnosis, and hospitalization outcomes can differ in these groups [8]. Because our cohort required a documented G30.x code, it represents hospitalizations in which AD was explicitly recorded and should not be interpreted as capturing all patients with underlying AD. This disease-specific approach improves etiologic clarity relative to an all-dementia cohort but may reduce sensitivity.

### 4.3. Why the Model A Versus Model B Framework Matters for Translation

The gain in discrimination from Model A to Model B should be interpreted according to prediction timing. Model A demonstrates that substantial mortality discrimination is achievable without explicit LOS, procedure counts, or charges. Model B performs better because it incorporates information that becomes available only as the hospital course unfolds. That improvement is therefore useful for dynamic reassessment but should not be presented as superior admission-time prediction. The finding that total charges were not independently significant in weighted regression and were not among the most stable top SHAP features further argues against treating charges as a direct severity measure.

This temporal separation also highlights an inherent limitation of administrative discharge data: although Model A excludes explicit inpatient-course utilization measures, diagnosis codes do not always establish when a condition became clinically apparent. Prospective or time-stamped electronic health record studies will be needed to validate truly admission-time versions of these models.

### 4.4. Explainability and Stability

SHAP was used here as a model-interpretation tool rather than evidence of causality [25,26,27]. The most valuable feature of the explainability analysis is the reproducibility of leading predictors across patient-grouped folds. Sepsis, diagnostic burden, acute kidney injury, and age remained central in Model A, while LOS and procedure burden emerged in Model B without displacing the principal acute illness signals. Presenting shared variables in a common order across Figure 3 panels improves visual comparison and makes the admission-versus-course distinction easier to interpret.

### 4.5. Diagnosis Family Context at LOS Extremes

The revised diagnosis family analysis provides clinical context without treating every coded condition as a causal determinant. Short stays were dominated by common chronic cardiometabolic diseases, whereas prolonged stays were enriched for electrolyte disturbances, dysphagia, delirium, infection, respiratory failure, acute kidney injury, and malnutrition-related diagnoses. Recent matched-cohort evidence also indicates that hospitalized patients with dementia have increased risks of delirium, pneumonia, falls, and pressure injury [6]. In our cohort, pressure ulcer coding was more than twice as prevalent in prolonged versus short stays. Prior geriatric hospital research has associated pressure ulcers with longer hospitalization [28]. Nevertheless, the NRD cannot reliably determine whether a pressure injury was present on admission or developed during hospitalization, so the observed enrichment should be interpreted as a marker of frailty and complex care rather than evidence that pressure ulcers caused prolonged LOS.

### 4.6. Limitations

This study has several limitations inherent to administrative data [29,30,31,32]. First, cohort identification depended on documentation of G30.x. Patients with clinically present but unrecognized, undocumented, or miscoded AD were not captured, and dementia under-recognition in routine care limits the sensitivity and generalizability of a code-defined cohort [8]. Second, the NRD lacks laboratory values, vital signs, medication data, detailed neurologic examinations, AD severity staging, frailty measures, functional status, and granular treatment timing. Diagnosis codes are discharge-level and cannot reliably distinguish pre-existing conditions from complications acquired during the hospitalization. Third, LOS is time-dependent and subject to survivorship dynamics and reverse causation; the analyses are associational and do not support interpreting LOS as a modifiable cause of mortality.

Fourth, total hospital charges are billing measures rather than standardized costs and vary with institutional charge structures, geography, service mix, and utilization; they should not be interpreted as direct measures of physiologic severity. Fifth, pressure injuries and other complications may influence and also result from prolonged hospitalization, but their temporal ordering cannot be established from the current coding. Sixth, the 2017 NRD reflects pre-pandemic practice. COVID-19 and subsequent changes in staffing, hospital capacity, infection burden, discharge pathways, and post-acute care may limit contemporary external validity [9,33]. Finally, the outcome was limited to in-hospital death and does not capture hospice transitions, deaths after discharge, readmission-related mortality, or longer-term functional outcomes.

### 4.7. Implications and Conclusion

The principal clinical implication is that LOS should not be interpreted in isolation. Very short hospitalizations identify a group with concentrated crude mortality, whereas the higher crude mortality observed in prolonged stays appears to be substantially explained by patient complexity and evolving inpatient-course factors. Admission-only models can support early risk communication and prioritization, but risk assessment should be updated as procedures, complications and other course information accumulate. In this full national G30.x cohort, patient-grouped explainable machine learning provided stable discrimination and interpretable predictors, while the revised spline analysis clarified that the unadjusted U-shaped LOS pattern does not persist in the same form after adjustment. Contemporary external validation in post-pandemic data is needed before clinical implementation.

## Figures and Tables

**Table 1:** Cohort characteristics by in-hospital mortality status (NRD 2017; Alzheimer’s disease hospitalizations, age ≥ 60 years).

Characteristic	Overall	Survived	Died In-Hospital	*p*-Value
Hospitalizations, n (unweighted)	249,507	236,841	12,666	—
In-hospital deaths, n (%) (unweighted)	12,666 (5.08)	—	—	—
National estimate, n (weighted)	455,256	432,644	22,612	—
In-hospital mortality, % (weighted)	4.97	—	—	—
Age, years (weighted mean)	—	82.39	83.95	<0.001
Number of diagnoses (weighted mean)	—	17.06	20.90	<0.001
Number of procedures (weighted mean)	—	0.86	1.83	<0.001
Length of stay, days (weighted median [IQR])	—	4 [3–7]	4 [2–9]	<0.001

Note: Descriptive national estimates use HCUP-NRD discharge weights (DISCWT). *p*-values compare survivors with decedents using unweighted two-sample *t* tests for continuous variables and Pearson χ^2^ tests for categorical variables; LOS was compared using a two-sided Wilcoxon rank-sum test. IQR, interquartile range; LOS, length of stay; NRD, Nationwide Readmissions Database.

**Table 2:** Weighted multivariable logistic regression for in-hospital mortality: Model A versus Model B.

Predictor	Model A OR (95% CI)	*p*-Value	Model B OR (95% CI)	*p*-Value
Primary payer: Medicaid (vs. Medicare)	1.29 (1.15–1.46)	<0.001	1.28 (1.13–1.45)	<0.001
Primary payer: Private insurance (vs. Medicare)	2.31 (2.18–2.45)	<0.001	2.41 (2.27–2.56)	<0.001
Primary payer: Self-pay (vs. Medicare)	1.99 (1.57–2.52)	<0.001	1.98 (1.55–2.53)	<0.001
Primary payer: No charge (vs. Medicare)	1.35 (0.45–4.06)	0.598	1.30 (0.45–3.74)	0.625
Primary payer: Other (vs. Medicare)	3.68 (3.39–3.99)	<0.001	3.89 (3.58–4.23)	<0.001
ZIP income quartile 2 (vs. quartile 1)	0.99 (0.95–1.03)	0.547	0.98 (0.94–1.01)	0.210
ZIP income quartile 3 (vs. quartile 1)	1.01 (0.97–1.06)	0.484	1.00 (0.96–1.05)	0.846
ZIP income quartile 4 (vs. quartile 1)	1.11 (1.06–1.16)	<0.001	1.10 (1.05–1.15)	<0.001
Urban–rural category 2 (vs. category 1)	0.90 (0.87–0.94)	<0.001	0.94 (0.90–0.98)	0.003
Urban–rural category 3 (vs. category 1)	0.97 (0.93–1.01)	0.103	0.99 (0.95–1.04)	0.762
Urban–rural category 4 (vs. category 1)	1.11 (1.05–1.17)	<0.001	1.16 (1.10–1.23)	<0.001
Urban–rural category 5 (vs. category 1)	1.20 (1.14–1.26)	<0.001	1.27 (1.20–1.34)	<0.001
Urban–rural category 6 (vs. category 1)	1.30 (1.23–1.38)	<0.001	1.38 (1.30–1.47)	<0.001
Age (per year)	1.04 (1.04–1.04)	<0.001	1.04 (1.04–1.04)	<0.001
Female sex (ref: male)	0.95 (0.92–0.98)	<0.001	0.94 (0.92–0.97)	<0.001
Sepsis	3.61 (3.50–3.73)	<0.001	3.59 (3.48–3.72)	<0.001
Acute kidney injury	1.90 (1.84–1.96)	<0.001	1.90 (1.84–1.96)	<0.001
Stroke	2.75 (2.61–2.90)	<0.001	2.84 (2.70–3.00)	<0.001
Number of diagnoses (I10_NDX)	1.05 (1.05–1.06)	<0.001	1.05 (1.05–1.06)	<0.001
Congestive heart failure	1.33 (1.28–1.37)	<0.001	1.34 (1.30–1.38)	<0.001
Urinary tract infection	0.66 (0.64–0.68)	<0.001	0.70 (0.68–0.72)	<0.001
Chronic kidney disease	0.79 (0.76–0.81)	<0.001	0.78 (0.76–0.81)	<0.001
COPD	0.93 (0.90–0.97)	<0.001	0.93 (0.90–0.97)	<0.001
Delirium	0.84 (0.79–0.89)	<0.001	0.96 (0.90–1.01)	0.119
Elective admission (ref: non-elective)	1.06 (0.98–1.14)	0.131	1.02 (0.95–1.10)	0.578
Pneumonia	1.55 (1.49–1.60)	<0.001	1.60 (1.54–1.66)	<0.001
Weekend admission (ref: weekday)	1.07 (1.04–1.11)	<0.001	1.06 (1.02–1.09)	<0.001
ED involvement (any evidence vs. none)	0.96 (0.91–1.01)	0.083	0.94 (0.90–0.99)	0.021
Number of procedures (I10_NPR)	—	—	1.18 (1.17–1.19)	<0.001
Total charges (per $10,000)	—	—	1.00 (1.00–1.00)	0.459

Note: ORs are weighted using DISCWT. Model A excludes explicit inpatient-course measures (LOS, procedure count, and total charges); Model B adds those measures. LOS in Model B was modeled using a restricted cubic spline with 5 degrees of freedom and therefore does not have a single OR. The overall LOS spline Wald test was *p* = 3.90 × 10^−187^, and the restricted-cubic-spline versus linear LOS likelihood-ratio test was *p* = 2.43 × 10^−256^. Reference categories are shown in the predictor labels. ZIP income quartile 1 represents ZIP codes with the lowest median household income, whereas quartile 4 represents ZIP codes with the highest median household income; CI, confidence interval; COPD, chronic obstructive pulmonary disease; ED, emergency department; OR, odds ratio.

## Data Availability

The data used in this study were obtained from the 2017 Healthcare Cost and Utilization Project (HCUP) Nationwide Readmissions Database (NRD), maintained by the Agency for Healthcare Research and Quality. Because the NRD is subject to the HCUP Data Use Agreement, the discharge-level data cannot be publicly shared or redistributed by the authors. Researchers may obtain access to the NRD directly from HCUP after completing the required training and agreeing to the applicable Data Use Agreement. The analysis code used in this study is available at the authors’ GitHub repository: https://github.com/TAlkam/NRD_2017 (accessed on 14 September 2026).

## Supplementary Materials

The following supporting information can be downloaded at: https://www.mdpi.com/article/10.3390/geriatrics11050136/s1, Figure S1: Decision curve analysis of patient-grouped out-of-fold mortality predictions; Table S1: Unadjusted in-hospital mortality across length-of-stay categories. Table S2: Sensitivity of model performance to restricted cubic spline degrees of freedom. Table S3: Threshold-dependent performance from patient-grouped out-of-fold predictions. Table S4: Fold-level stability of SHAP feature importance. Table S5: Decision curve net benefit across threshold probabilities. Table S6A: Top diagnosis families among short length-of-stay hospitalizations (LOS ≤ 5 days; N = 152,991). Table S6B: Top diagnosis families among prolonged length-of-stay hospitalizations (LOS ≥ 25 days; N = 6212). Table S6C: Diagnosis families most enriched in prolonged versus short length-of-stay hospitalizations. Table S7: Regularized logistic-regression comparator under patient-grouped out-of-fold evaluation.
