# Supplementary material for: Nonlinear Association of Length of Stay with In-Hospital Mortality in Alzheimer’s Disease Hospitalizations: Admission-Only vs. Inpatient-Course Prediction Using Explainable Machine Learning
Source: Geriatrics (Basel). 2026 Sep 17;11(5):136. doi: 10.3390/geriatrics11050136 (PMC13600026; doi:10.3390/geriatrics11050136)
Supplement: Supplementary file 1 [file geriatrics-11-00136-s001.zip › geriatrics-4435428-supplementary.pdf]

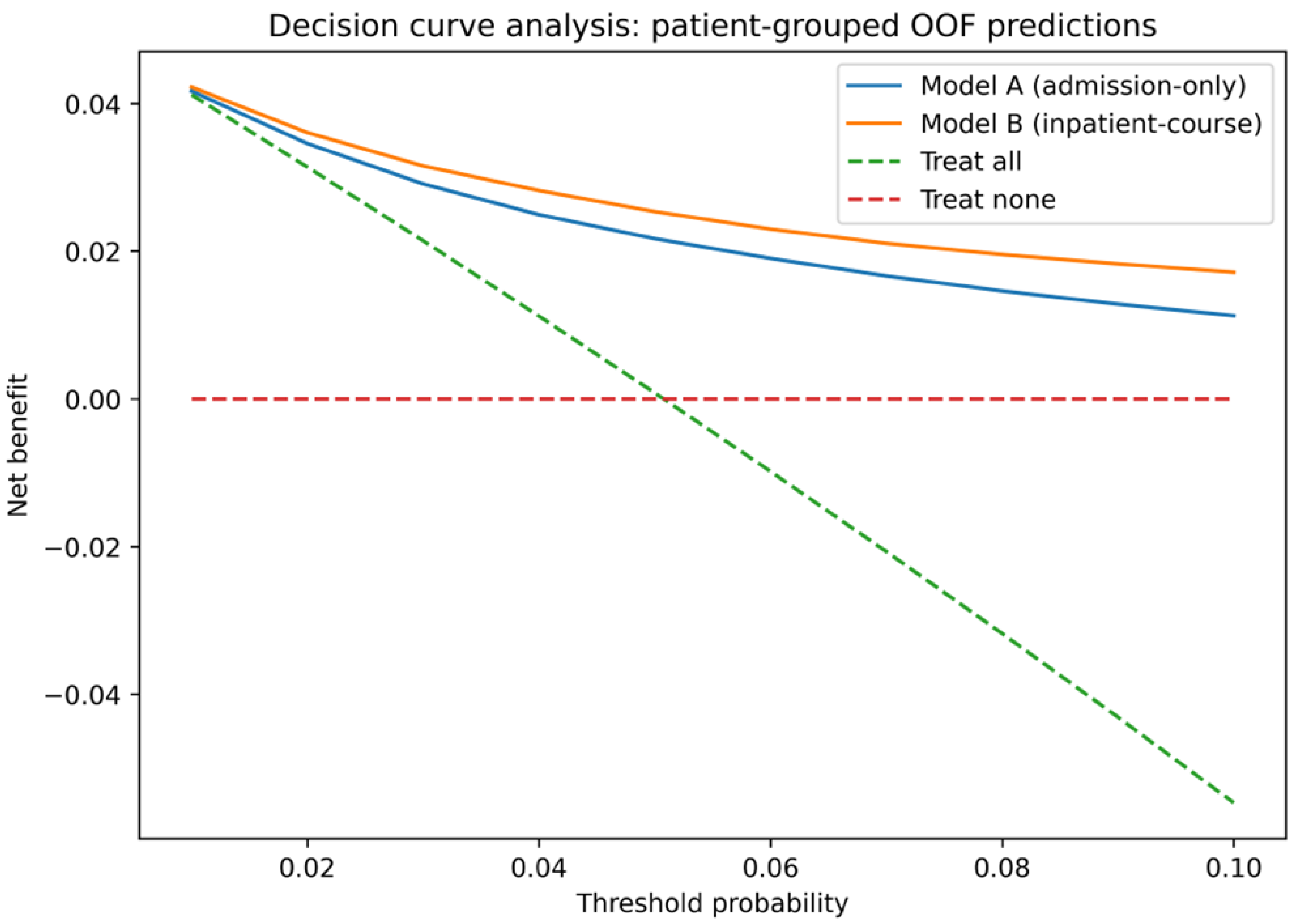

Supplementary Figure S1

Supplementary Figure S1. Decision curve analysis of patient-grouped out-of-fold mortality predictions. Decision curve analysis compares the clinical net benefit of the admission-oriented Model A and the full inpatient-course Model B across threshold probabilities from 0.01 to 0.10 using patient-grouped out-of-fold predictions. The dashed reference lines represent the default strategies of treating all patients and treating none. Both models provided greater net benefit than the treat-all and treat-none strategies across the evaluated threshold range,

and Model B consistently provided higher net benefit than Model A. These findings suggest greater potential clinical utility when inpatient-course information is incorporated into the prediction framework.

Supplementary Table S1. Unadjusted in-hospital mortality across length-of-stay categories.

LOS, days	Hospitalizations, n	Deaths, n	Mortality, % (95% CI)	Admissions, %	All deaths, %
0–1	20,221	2,629	13.00 (12.54–13.47)	8.11	20.76
2–3	73,238	2,738	3.74 (3.60–3.88)	29.36	21.62
4–6	78,887	2,739	3.47 (3.35–3.60)	31.63	21.62
7–9	33,968	1,649	4.85 (4.63–5.09)	13.62	13.02
10–14	22,922	1,418	6.19 (5.88–6.51)	9.19	11.20
15–21	11,723	803	6.85 (6.41–7.32)	4.70	6.34
≥22	8,479	659	7.77 (7.22–8.36)	3.40	5.20

Note: LOS-dependent analyses included 249,438 hospitalizations with non-missing LOS and 12,635 in-hospital deaths. Percent of all deaths uses the LOS-complete death count as the denominator. CI, confidence interval; LOS, length of stay.

Supplementary Table S2. Sensitivity of model performance to restricted cubic spline degrees of freedom.

Spline df	Pooled OOF AUROC	Fold AUROC, mean ± SD	Pooled OOF AUPRC	Pooled OOF log loss
4	0.785	0.785 ± 0.002	0.204	0.172
5	0.785	0.785 ± 0.002	0.206	0.172
6	0.786	0.786 ± 0.002	0.207	0.171

Note: Performance was evaluated using leakage-resistant patient-grouped out-of-fold predictions. The 5-degree-of-freedom specification was prespecified for the primary analysis. AUPRC, area under the precision–recall curve; AUROC, area under the receiver operating characteristic curve; df, degrees of freedom; OOF, out-of-fold; SD, standard deviation.

Supplementary Table S3. Threshold-dependent performance from patient-grouped out-of-fold predictions.

Model	Threshold label	Threshold	Precision	Recall	F1	Flagged, %	TN	FP	FN	TP
Model A	Best F1	0.131	0.202	0.358	0.259	8.98	218,963	17,878	8,132	4,534
Model A	0.05	0.050	0.120	0.698	0.204	29.62	171,790	65,051	3,820	8,846
Model A	0.10	0.100	0.173	0.468	0.253	13.74	208,499	28,342	6,738	5,928
Model A	0.15	0.150	0.220	0.303	0.255	7.00	223,219	13,622	8,829	3,837
Model A	0.20	0.200	0.256	0.198	0.223	3.92	229,574	7,267	10,159	2,507
Model A	0.50	0.500	0.448	0.006	0.013	0.07	236,741	100	12,585	81
Model B	Best F1	0.164	0.332	0.382	0.355	5.85	227,080	9,761	7,824	4,842
Model B	0.05	0.050	0.144	0.727	0.240	25.67	182,010	54,831	3,456	9,210
Model B	0.10	0.100	0.232	0.531	0.323	11.63	214,552	22,289	5,945	6,721
Model B	0.15	0.150	0.310	0.407	0.352	6.67	225,345	11,496	7,513	5,153
Model B	0.20	0.200	0.376	0.321	0.346	4.33	230,106	6,735	8,605	4,061
Model B	0.50	0.500	0.695	0.118	0.202	0.86	236,185	656	11,172	1,494

Note: Metrics were calculated from patient-grouped out-of-fold predicted probabilities. “Best F1” denotes the threshold maximizing F1 in the out-of-fold predictions and should not be interpreted as a prespecified clinical cutoff. F1, harmonic mean of precision and recall; FN, false negative; FP, false positive; TN, true negative; TP, true positive.

Supplementary Table S4. Fold-level stability of SHAP feature importance.

Model	Predictor	Top-10 folds	Average rank when in top 10	Mean |SHAP|
Model A	Sepsis	5/5	1.0	0.498
Model A	Number of diagnoses (ICD-10-CM)	5/5	2.0	0.377
Model A	Acute kidney injury	5/5	3.0	0.238
Model A	Age (years)	5/5	4.0	0.210
Model A	Urinary tract infection	5/5	5.0	0.161
Model A	Pneumonia	5/5	6.0	0.127
Model A	Stroke	5/5	7.4	0.122
Model A	Primary payer	5/5	7.8	0.120
Model A	Congestive heart failure	5/5	8.8	0.114
Model A	Chronic kidney disease	4/5	10.0	0.088
Model A	Urban–rural category	1/5	10.0	0.096
Model B	Sepsis	5/5	1.0	0.460
Model B	Number of diagnoses (ICD-10-CM)	5/5	2.0	0.393
Model B	Length of stay (days)	5/5	3.0	0.317
Model B	Number of procedures	5/5	4.0	0.262
Model B	Acute kidney injury	5/5	5.0	0.239

Model	Predictor	Top-10 folds	Average rank when in top 10	Mean |SHAP|
Model B	Age (years)	5/5	6.0	0.233
Model B	Pneumonia	5/5	7.0	0.145
Model B	Stroke	5/5	9.2	0.122
Model B	Primary payer	4/5	8.5	0.125
Model B	Urinary tract infection	3/5	8.7	0.122
Model B	Congestive heart failure	3/5	9.7	0.121

Note: Within each patient-grouped validation fold, predictors were ranked by mean absolute SHAP value. “Top-10 folds” is the number of the five folds in which a predictor appeared among the 10 highest-ranked features. Mean |SHAP| is summarized across folds in which the predictor appeared in the top 10. SHAP, SHapley Additive exPlanations.

Supplementary Table S5. Decision curve net benefit across threshold probabilities.

Threshold probability	Model A	Model B	Treat all	Treat none
0.01	0.0417	0.0422	0.0412	0.0000
0.02	0.0345	0.0360	0.0314	0.0000
0.03	0.0291	0.0316	0.0214	0.0000
0.04	0.0250	0.0281	0.0112	0.0000
0.05	0.0217	0.0253	0.0008	0.0000
0.06	0.0189	0.0230	-0.0098	0.0000
0.07	0.0165	0.0209	-0.0207	0.0000
0.08	0.0147	0.0195	-0.0318	0.0000
0.09	0.0129	0.0182	-0.0431	0.0000
0.10	0.0111	0.0170	-0.0547	0.0000

Note: Net benefit was calculated from patient-grouped out-of-fold predicted probabilities. Model B showed greater net benefit than Model A at each evaluated threshold from 0.01 to 0.10. The treat-none strategy has net benefit 0 by definition.

Supplementary Table S6A. Top diagnosis families among short length-of-stay hospitalizations (LOS ≤5 days; N=152,991).

ICD-10-CM family	Diagnosis family	Hospitalizations, n	Prevalence, %
E78	Hyperlipidemia	71,310	46.61
I10	Hypertension	67,787	44.31
E11	Type 2 diabetes mellitus	45,025	29.43
E87	Fluid/electrolyte disorders	44,676	29.20
I25	Chronic ischemic heart disease	42,192	27.58
N18	Chronic kidney disease	38,568	25.21
I48	Atrial fibrillation/flutter	37,662	24.62
N39	Urinary tract infection	37,575	24.56
K21	Gastroesophageal reflux disease	34,614	22.62
N17	Acute kidney injury	34,466	22.53
E86	Volume depletion	33,398	21.83
E03	Other hypothyroidism	32,835	21.46
I50	Heart failure	32,555	21.28
F32	Major depressive disorder, single episode	26,308	17.20
J44	Chronic obstructive pulmonary disease	24,992	16.34
G93	Other disorders of brain	24,303	15.89

ICD-10-CM family	Diagnosis family	Hospitalizations, n	Prevalence, %
I12	Hypertensive chronic kidney disease	22,249	14.54
A41	Sepsis	20,534	13.42
F41	Other anxiety disorders	19,959	13.05
D64	Other anemias	17,774	11.62
M19	Other and unspecified osteoarthritis	17,561	11.48
J96	Respiratory failure	16,845	11.01
N40	Benign prostatic hyperplasia	16,224	10.60
I11	Hypertensive heart disease	16,012	10.47
B96	Bacterial agents as cause of diseases classified elsewhere	15,579	10.18

Note: Each three-character ICD-10-CM diagnosis family was counted at most once per hospitalization. G30, F01-F03, Z-code families, and external-cause families beginning with V, W, X, or Y were excluded to emphasize clinical diagnosis families rather than cohort-defining, dementia, administrative, or external-cause codes. LOS, length of stay.

Supplementary Table S6B. Top diagnosis families among prolonged length-of-stay hospitalizations (LOS ≥25 days; N=6,212).

ICD-10-CM family	Diagnosis family	Hospitalizations, n	Prevalence, %
I10	Hypertension	3,006	48.39
E87	Fluid/electrolyte disorders	2,875	46.28
E78	Hyperlipidemia	2,752	44.30
N39	Urinary tract infection	2,151	34.63
N17	Acute kidney injury	1,934	31.13
E11	Type 2 diabetes mellitus	1,929	31.05
I25	Chronic ischemic heart disease	1,612	25.95
N18	Chronic kidney disease	1,547	24.90
K21	Gastroesophageal reflux disease	1,469	23.65
G93	Other disorders of brain	1,462	23.54
I48	Atrial fibrillation/flutter	1,457	23.45
E86	Volume depletion	1,423	22.91
F32	Major depressive disorder, single episode	1,343	21.62
I50	Heart failure	1,331	21.43
J96	Respiratory failure	1,293	20.81
F41	Other anxiety disorders	1,285	20.69

ICD-10-CM family	Diagnosis family	Hospitalizations, n	Prevalence, %
R13	Dysphagia	1,240	19.96
E03	Other hypothyroidism	1,235	19.88
A41	Sepsis	1,217	19.59
D64	Other anemias	1,200	19.32
K59	Other functional intestinal disorders	1,108	17.84
J44	Chronic obstructive pulmonary disease	1,087	17.50
F05	Delirium due to known physiological condition	1,034	16.65
I12	Hypertensive chronic kidney disease	941	15.15
B96	Bacterial agents as cause of diseases classified elsewhere	896	14.42

Note: Each three-character ICD-10-CM diagnosis family was counted at most once per hospitalization. G30, F01-F03, Z-code families, and external-cause families beginning with V, W, X, or Y were excluded. LOS, length of stay.

Supplementary Table S6C. Diagnosis families most enriched in prolonged versus short length-of-stay hospitalizations.

ICD-10-CM family	Diagnosis family	Short LOS ≤5 days, n (%)	Prolonged LOS ≥25 days, n (%)	Difference, percentage points
E87	Fluid/electrolyte disorders	44,676 (29.20)	2,875 (46.28)	17.08
R13	Dysphagia	12,369 (8.08)	1,240 (19.96)	11.88
F05	Delirium due to known physiological condition	7,982 (5.22)	1,034 (16.65)	11.43
K59	Other functional intestinal disorders	9,957 (6.51)	1,108 (17.84)	11.33
N39	Urinary tract infection	37,575 (24.56)	2,151 (34.63)	10.07
J96	Respiratory failure	16,845 (11.01)	1,293 (20.81)	9.80
N17	Acute kidney injury	34,466 (22.53)	1,934 (31.13)	8.61
L89	Pressure ulcer	8,659 (5.66)	849 (13.67)	8.01
J69	Pneumonitis due to solids and liquids	8,828 (5.77)	843 (13.57)	7.80
D64	Other anemias	17,774 (11.62)	1,200 (19.32)	7.70
R45	Symptoms involving emotional state	2,027 (1.32)	558 (8.98)	7.66
G93	Other disorders of brain	24,303 (15.89)	1,462 (23.54)	7.65
F41	Other anxiety disorders	19,959 (13.05)	1,285 (20.69)	7.64
E43	Severe protein-calorie malnutrition	5,842 (3.82)	665 (10.71)	6.89
G47	Sleep disorders	10,191 (6.66)	815 (13.12)	6.46
A41	Sepsis	20,534 (13.42)	1,217 (19.59)	6.17

ICD-10-CM family	Diagnosis family	Short LOS ≤5 days, n (%)	Prolonged LOS ≥25 days, n (%)	Difference, percentage points
E83	Disorders of mineral metabolism	7,901 (5.16)	699 (11.25)	6.09
B37	Candidiasis	1,548 (1.01)	407 (6.55)	5.54
F22	Delusional disorders	674 (0.44)	361 (5.81)	5.37
R53	Malaise and fatigue	9,651 (6.31)	721 (11.61)	5.30
E46	Unspecified protein-calorie malnutrition	3,616 (2.36)	457 (7.36)	4.99
R62	Lack of expected normal physiological development	7,886 (5.15)	630 (10.14)	4.99
R65	Systemic inflammatory response syndrome	9,142 (5.98)	677 (10.90)	4.92
E55	Vitamin D deficiency	4,651 (3.04)	465 (7.49)	4.45
F32	Major depressive disorder, single episode	26,308 (17.20)	1,343 (21.62)	4.42

Note: The table presents the 25 diagnosis families with the largest absolute prevalence enrichment in prolonged stays, calculated as prevalence in LOS ≥25 days minus prevalence in LOS ≤5 days. Group denominators were 6,212 and 152,991, respectively. Each diagnosis family was counted at most once per hospitalization; G30, F01-F03, Z-code families, and V/W/X/Y external-cause families were excluded. LOS, length of stay.

Supplementary Table S7. Regularized logistic-regression comparator under patient-grouped out-of-fold evaluation.

Model	N	Deaths	Prevalence, %	Pooled OOF AUROC	Pooled OOF AUPRC	Fold AUROC, mean ± SD	Fold AUPRC, mean ± SD
Model A (admission-only)	249,507	12,666	5.08	0.776	0.175	0.776 ± 0.000	0.175 ± 0.002
Model B (inpatient-course)	249,507	12,666	5.08	0.784	0.193	0.784 ± 0.002	0.194 ± 0.004

Note: Regularized logistic regression was evaluated using the same patient-grouped 5-fold out-of-fold framework used for the primary machine-learning models. AUPRC, area under the precision–recall curve; AUROC, area under the receiver operating characteristic curve; OOF, out-of-fold; SD, standard deviation.
